# Healthcare Providers’ Perceptions and Experiences of Prenatal Iron and Folic Acid Supplementation—A Qualitative Study in Botswana

**DOI:** 10.1016/j.cdnut.2026.107660

**Published:** 2026-02-25

**Authors:** Letumile Moeng, Poloko Kebaabetswe, Modiegi Diseko, Rebecca Zash, Gloria Mayondi, Judith Mabuta, Mompati Mmalane, Joseph Makhema, Shahin Lockman, Elizabeth Lowenthal, Roger Shapiro, Ellen C Caniglia

**Affiliations:** 1 Botswana-Harvard Health Partnership, Gaborone, Botswana; 2 Internal Medicine-Infectious Disease, Beth Israel Deaconess Medical Center, Boston, MA, United States; 3 Immunology and Infectious Disease, Harvard T.H. Chan School of Public Health, Boston, MA, United States; 4 Infectious Disease, Brigham and Women’s Hospital, Boston, MA, United States; 5 Department of Biostatistics, Epidemiology, and Informatics, University of Pennsylvania Perelman School of Medicine, Philadelphia, PA, United States

**Keywords:** iron, folic acid, prenatal vitamin supplementation, multiple micronutrient supplementation, barriers and facilitators, qualitative interviews

## Abstract

**Background:**

The World Health Organization recommends daily iron and folic acid (IFA) supplementation during pregnancy to prevent maternal anemia and adverse birth outcomes such as preterm birth. However, uptake of IFA has varied substantially in low- and middle-income countries due to issues regarding supply, knowledge, access to health services, and acceptability. In Botswana, nearly one-third of pregnant women do not receive IFA. **Objectives:** To assess knowledge of IFA supplementation, supplement availability, and barriers and facilitators to preconception and antenatal supplementation in Botswana.

**Methods:**

We conducted qualitative interviews with 2 key stakeholder groups at 2 different levels: the individual level (pregnant women) and the service delivery level (healthcare providers). In this study, we present results from 16 interviews with healthcare providers at 2 representative antenatal clinic sites in Botswana in 2022—8 nurse/midwives, 4 pharmacists, 2 dieticians, and 2 Ministry of Health sexual and reproductive health officials.

**Results:**

Healthcare providers were knowledgeable about the benefits of IFA supplementation and prescribed supplements when available. Several key barriers were identified: lack of availability of supplements due to frequent and long-lasting stockouts, late antenatal care registration, nonadherence and side effects, costs, and cultural and traditional beliefs. Healthcare providers indicated that foods rich in IFA were available and that fortification of staple foods with IFA would be feasible and acceptable.

**Conclusions:**

Our study identified an urgent need to increase the availability of IFA supplementation at antenatal clinics, with the ultimate goal of improving maternal and infant outcomes. In addition, addressing patient concerns around side effects and providing foods fortified with IFA to pregnant women could improve maternal and infant health.

## Introduction

Reducing preterm delivery (PTD), small for gestational age (SGA) births, and stillbirths (SB) is a priority for the WHO and United Nations’ Every Newborn Action Plan. Small babies have the highest risk of death in utero, during the neonatal period and throughout early childhood, and preterm delivery is the leading cause of death among children aged <5 y [1]. The risk of adverse birth outcomes is high in Botswana (∼30%) [2], especially among the 23% of women living with HIV, [3] and there is an urgent need for scalable, low-cost interventions to improve maternal and infant health.

Maternal anemia is a well-established risk factor for PTD, SGA, and SB globally and in Botswana [4], where the prevalence of anemia among pregnant women has remained stagnant at 31% from 2010 to 2019 [5]. Iron deficiency is the most common cause of anemia globally [6,7], but at least half of anemia cases are attributable to other causes, including deficiencies in micronutrients (e.g., folic acid) and infectious diseases (e.g., HIV and malaria) [[8], [9], [10], [11], [12], [13]]. To address these risks, WHO recommends daily supplementation with iron (30–60 mg) and folic acid (0.4 mg) (IFA) during pregnancy to prevent PTD, SGA, maternal anemia, and sepsis [14].

Most recently, evidence has suggested that multiple micronutrient supplementation (MMS), which includes IFA plus additional micronutrients, may further reduce the risk of adverse pregnancy outcomes [[15], [16], [17]]. In 2020, WHO updated its guidance to recommend MMS during pregnancy in the context of rigorous research, highlighting the need for additional implementation research to inform programmatic scale-up [18].

Despite longstanding WHO recommendations on IFA, significant gaps in the implementation of IFA strategies persist globally. IFA supplementation is standard of care in Botswana and supplements are prescribed by clinic pharmacies at no cost to pregnant women; however, approximately one-third of pregnant women receiving antenatal services do not receive guideline-concordant supplementation [19]. In addition, staple foods are not routinely fortified with micronutrients [20,21] and there is currently no mandatory national folic acid fortification program.

Understanding barriers to prenatal supplementation implementation is therefore critical for strengthening existing IFA programs and informing the effective implementation of MMS. Prior studies across low- and middle-income countries have identified multiple barriers to IFA supplementation during pregnancy, including challenges related to supply and distribution, acceptability and side effects, inadequate counseling and knowledge, and limited access to and utilization of antenatal care services [[22], [23], [24], [25], [26], [27], [28], [29]]. Our prior qualitative work among pregnant women in Botswana found that supply constraints and education were perceived as the most important barriers to supplementation during pregnancy [30]. However, perspectives from healthcare providers, who are central to prescribing, counseling, procuring, and distributing supplements, are needed. Furthermore, improving maternal micronutrient status may also require complementary strategies such as preconception supplementation, food fortification, and improvements in dietary intake of foods rich in iron and folate.

To address this gap, we conducted in-depth qualitative interviews with healthcare providers in Botswana to explore their perceptions and experiences of prenatal supplementation during the preconception and antenatal periods, as well as dietary intake of foods rich in iron and folate and food fortification. In alignment with WHO’s call for rigorous implementation research to support MMS delivery, this study generates evidence on provider-level barriers to supplementation that are central to both existing IFA programs and future MMS scale-up.

## Methods

### Design and methods

#### Study design

The study employed a nonrandomized purposive qualitative design to enable the researchers to assess barriers and facilitators to IFA supplementation during and before pregnancy, as well as dietary intake of foods rich in iron and folate and food fortification. The overall study focused on 2 key stakeholder groups at 2 different levels: the individual level (pregnant women) and the service delivery level (healthcare providers). Healthcare providers included nurses/midwives, pharmacists, dieticians, and Ministry of Health officers in the sexual and reproductive health department. The specific objectives of the study for the service delivery level were: *1*) to assess healthcare providers’ knowledge of IFA supplementation benefits and foods rich in vitamins and micronutrients; *2*) to understand the availability of supplements at the clinic and national level in Botswana; *3*) to identify competing priorities and barriers to supplementation across the preconception and antenatal periods; and *4*) to ascertain factors that could facilitate preconception and prenatal supplementation.

#### Study sites

The study was conducted at 2 antenatal clinics, 1 in the Southern (Tlokweng) and another in the Northern (Palapye) region of Botswana, selected as diverse clinics serving a population representative of pregnant women seeking antenatal care throughout Botswana. The Kediretswe clinic in Palapye serves pregnant women from rural villages surrounding Palapye, whereas the Tlokweng main clinic serves those from semiurban and urban environments near the capital city of Gaborone. Site selection was facilitated by the District Health Management Team (DHMT). The DHMT was asked to guide the team toward large antenatal care sites, serving diverse and representative populations. The clinics selected provide antenatal care to >80 pregnant women each month.

#### Recruitment and consent

Healthcare providers were recruited through snowball sampling, a recruitment technique in which participants are asked to assist the research team in identifying other potential participants. Specifically, the DHMT managers directed the research team to the clinic leadership at each of the selected clinics, who then provided a list of the healthcare providers at the clinic. Healthcare providers were then contacted by the study team, and those who were recruited also provided names of those who could participate. The research assistants contacted individuals to inform them about the study and ask them if they were willing to participate. The consent process described the purpose of the study and the reason for the information being collected. Participants were informed that they could decline or withdraw participation at any point during the interview. The consent conversation took place in either Setswana or English, according to the preference of the individual participant. All participants signed a consent form to participate and for the interview to be audio recorded.

#### Study population and sample size

Recruitment took place between January and March 2022. The target enrollment included 8 nurses or midwives, 4 pharmacists, 2 dietitians, and 2 reproductive health officers. The research team aimed at collecting information from different cadres of healthcare providers to make sure that a variety of perspectives were represented. This sample size was hypothesized to be sufficient to reach thematic saturation and was guided by the concept of information power, suggesting that the different cadres selected purposefully by virtue of their profession and experience would provide rich and relevant information.

#### Data collection

A semistructured interview guide was developed by the research team based on the objectives of the study (Supplementary Material). Data collection employed a face-to-face in-depth interview. Eligible participants were asked to participate in an interview at the clinic or at their preferred place and at a convenient time. Two research assistants were hired and trained on the protocol: interview guides and ethics. The interviews were conducted by the local research assistants in English or Setswana. Interviews were conducted in a private location and audio recorded and lasted between 30 and 45 min. Interview recordings were first transcribed and then translated into English by the study coordinator.

The key interview domains are shown in Table 1 and included: *1*) provider knowledge of benefits of supplements, *2*) provider prescribing of IFA, *3*) availability of supplements at the clinic pharmacy and national level, *4*) major challenges to daily supplementation (including side effects and availability), *5*) availability of foods rich in iron and folate and food fortification and *6*) supplementation before pregnancy. The first 2 domains were addressed via questions to nurses and midwives only, whereas domains 3–6 were addressed via questions to all healthcare providers.

**TABLE 1:** Key interview domains and corresponding interview questions

Domain	Question stems
Provider knowledge of benefits of supplements (N/M) only)	What are some of the benefits of supplementation with IFA during pregnancy? Do you discuss IFA supplementation with a pregnant woman at her first antenatal visit? If so, what information do you provide? Do you discuss this at the first visit only, or at each visit during pregnancy? Do you discuss foods that are rich in IFA with pregnant women at their antenatal clinic visits? (Give examples).
Provider prescribing of IFA (N/M only)	Do you prescribe supplements with IFA to pregnant women? If yes, which ones? When in pregnancy do you prescribe them? If no, why not? If you only prescribe to some women, which women? Pregnant women are often prescribed iron alone, iron + folate, or a multiple micronutrient containing iron, folate and other micronutrients. What are the reasons why you would prescribe one versus another type of supplement? Do you prescribe supplements to women of child-bearing age who are not pregnant? Why or why not? If yes, which supplements?
Availability of supplements at clinic pharmacy and national level	In your experience, what supplements are available for pregnant women at antenatal clinic pharmacies? Which supplements are usually available and which are usually out of stock? If supplements are not available, do you know why? In your experience, what supplements are available at the national level? What other issues are there with supply and demand of supplements? Do you know when/where/how often supplements are available? If they are not available, do you know why?
Major challenges to supplementation (competing priorities, cost, side effects, availability, and knowledge)	In your experience, what are the major challenges to daily supplementation during pregnancy in Botswana (probe for cost, other priorities, other reasons)? Are there any side effects of supplements? If so, which ones? In your opinion, are supplements accessible or not accessible to pregnant women in Botswana? Give reasons for your response. Are pregnant women knowledgeable or not knowledgeable about the benefits of supplementation? Give reasons for your response. What are some approaches to overcoming these challenges? What are the major challenges to daily supplementation before pregnancy? In your experience, what are other barriers to supplementation with IFA during pregnancy? Before pregnancy? (N/M only) Are there side effects from supplementation with IFA? If so, what are they? Do you discuss these with pregnant women? (N/M only) To your knowledge, how can side effects be reduced? Do you discuss this with pregnant women?
Availability of foods rich in iron and folate and food fortification	How available are foods rich in IFA in Botswana? (Give examples). How does this vary by region or other factors? In your experience, how feasible/acceptable is fortification of staple foods with IFA in Botswana as a strategy for improving supplementation for women of child-bearing age? (Give examples). What are the key foods that could be fortified?
Supplementation before pregnancy	In your experience, how feasible/acceptable/accessible is supplementation before pregnancy in Botswana? Does this vary by region or other factors? How common is it that a woman you see for the first time has been taking supplements at least somewhat regularly since she became pregnant? What about before pregnancy?

Abbreviations: IFA, iron and folic acid; N/M, nurses and midwives.

Complete interview guides, including question probes, are available in the Suppementary Material.

N/M only indicates these questions were asked to N/M only. All other questions were asked to all healthcare providers.

#### Data management and quality

The study coordinator met with the research assistants before the interviews began to review the interview guides and approaches and was present during the first interviews at each site to ensure that interviewers were aligned in their approaches. At the end of each interview, the recorded data were forwarded to the study coordinator, who listened to the recordings and provided feedback to the research assistants on areas where improvements were needed (e.g., failure to probe).

#### Ethical considerations

All participants provided informed consent for study participation. Participants were given the liberty to not answer questions they did not feel comfortable answering and could withdraw their participation at any point during the interview.

The study was approved by the Institutional Review Board at the University of Pennsylvania Perelman School of Medicine and by the Human Research Development Council (HRDC) in Botswana. All personnel involved in the conduct of this study had completed Human Subjects Protection Training. The study coordinator made sure that, throughout the study, all relevant ethical principles for conducting interviews with the relevant stakeholders were observed. Key ethical principles observed by the research team included being sensitive to beliefs, manners, and customs of participants, acting with integrity and honesty with participants, ensuring a respectful communication and contact with participants, protecting the anonymity and confidentiality of individual information, and obtaining informed consent from everyone interviewed. Participants were given the liberty to not answer questions they did not feel comfortable answering and could withdraw their participation at any point during the interview.

### Data analysis

Interviews were transcribed verbatim and translated into English. Two members of the study team (PK and EC), both familiar with the study context and research questions, reviewed all English transcripts to gain a better understanding of the content before data analysis. Duplicate translation was not conducted. Data analysis followed a conventional content analysis approach, in which themes and codes were derived inductively from the data to identify key insights, common patterns, and shared experiences [31,32]. Major themes and codes were identified by examining trends, patterns, and ideas that appeared repeatedly throughout the data. Using ATLAS.ti, 1 member of the study team (PK) conducted the initial coding of all transcripts. An initial codebook was developed iteratively through close readings of the transcripts, and relationships between codes and themes were summarized in an overview table. Coded data, emerging themes, and interpretations were discussed with a second team member (EC), who reviewed coding decisions and thematic summaries. No new themes emerged in later interviews, suggesting saturation across provider cadres and sites.

## Results

A total of 16 healthcare providers participated in the study: 8 nurse/midwives (4 in Tlokweng and 4 in Palapye), 4 pharmacists (2 in Tlokweng and 2 in Palapye), 2 dietitians (both in Palapye), and 2 Ministry of Health sexual and reproductive health officers (1 in Tlokweng and 1 in Palapye).

### Domain 1: provider knowledge and education of benefits of IFA supplements

Nurse/midwives were asked about their knowledge of the benefits of IFA supplementation. Nurses and midwives demonstrated knowledge of the benefits of supplementation, for example, all (8/8) indicated that iron prevents anemia and most (7/8) indicated that folic acid prevents neural tube defects such as spina bifida. In addition to IFA, they also indicated that vitamin C could be given to pregnant women to facilitate absorption of supplements. Nurse/midwives also indicated that they provide information about the benefits of IFA supplementation and foods rich in iron and folate to pregnant women starting at the first antenatal visit.

*Usually we provide them with information on the importance and benefits of supplements and how they are to be taken. You do not take them alone. You accompany them with healthy eating. – Nurse/midwife*

### Domain 2: provider prescribing of iron and folic acid

Nurse/midwives were asked about their practices prescribing supplements with IFA to pregnant women and women of child-bearing age. Nurse/midwives described prescribing IFA, both as separate tablets or in combination, as well as calcium and multivitamins (containing IFA) to pregnant women. Nurses highlighted that supplements were usually prescribed during the first antenatal visit (in Botswana, the majority of women first engage in prenatal care in the 2nd trimester [19]). When asked about supplement preferences, nurse/midwives indicated that their preference was to prescribe all pregnant women with IFA, but that in practice decisions about which supplements to prescribe were based almost exclusively on availability. One nurse/midwife mentioned that combination pills may improve adherence.

*We normally prescribe according to availability, the one that is available we give. If we have iron with folic, we prescribe that, and if we have iron alone, we will prescribe iron alone, folic alone and other multivitamins or vitamin C, but we depend entirely on the availability. – Nurse/midwife*

*I think when looking at those drugs I would prescribe iron and folic acid because this combination when one is taking it the compliance would be much easier compared to prescribing folic alone or iron alone. – Nurse/midwife*

When asked about prescribing supplements to nonpregnant women of child-bearing potential, nurse/midwives said that pregnant women were prioritized for receiving supplements. Supplements were sometimes prescribed to nonpregnant women who indicated that they wanted to conceive or had health complications such as low hemoglobin, but this practice was inconsistent.

*To be honest we do not [prescribe supplements to nonpregnant women], which is wrong because the clients we see are women who are pregnant already or women who are here for family planning. And those here for family planning, for us to prescribe ferrous is only when they have complications like spotting due to the contraceptives they are using, but most women do not come to the clinic even though we teach them during family planning that when they plan to conceive, they must come and let us know so that we give the supplements. – Nurse/midwife*

### Domain 3: availability of supplements at the clinic pharmacy and national level

All participants were asked about the availability of supplements at the clinic and national levels. One of the dietitians indicated that IFA is not readily available due to stockouts, and that some pregnant women do not present for care early enough to receive supplementation. The reproductive health officers also noted the frequent stockouts of supplements, with 1 noting that vitamin C was the only currently available micronutrient and the other noting that only folic acid was currently available. Pharmacists echoed the issues with stockouts, with 1 noting a particularly long period in which supplements were not available.

*It’s been long, over a year [since we had ferrous and folic acid]. We once received some, but it was not enough – Pharmacist*

The nurse/ midwives also described that supplements were often not available due to stockouts at Central Medical Stores, the national-level government supplier of medicines and supplements in Botswana. Nurse/midwives raised the concern that frequent stockouts negatively affected pregnant women who needed supplements the most, and that stockouts sometimes lasted 6 mo. They stated that supplements were recommended immediately at antenatal registration but that, due to frequent and lengthy stockouts, some pregnant women were instead provided with nutrient fortified foods like Tsabana and Malutu (a mixture of sorghum and soya [33]), and encouraged to eat foods rich in iron and folate such as vegetables, fruits, nuts, eggs, liver and locally available foods.

*Currently we do not have most supplements, we only have multi-vitamin, we do not have ferrous sulfate [iron] and folic, it has been like this for the past 3 months, even at the local pharmacies it has been exhausted. It is out of stock from the central medical stores – Nurse/midwife*

### Domain 4: major challenges to daily supplementation

All healthcare providers were asked about major challenges to daily supplementation during pregnancy. The most frequently mentioned challenges were availability of supplements (stockouts), competing priorities, late antenatal care registration, nonadherence and side effects, costs, and cultural and traditional beliefs.

#### Availability of supplements (stockouts)

The most frequently noted challenge was the availability of supplements, with most participants indicating that the availability of supplements was a substantial challenge and that lack of availability in health facilities negatively affected pregnant women. Inconsistent supply was also noted, where supplements were available briefly in some clinics but then were not available for a long time. The effect of stockouts was more pronounced in remote villages where there is often only 1 clinic within traveling distance.

*If the pregnant woman is at Moiyabana or Tshimoyapula, her access to the supplementation there is different to when she is in Serowe, it affects whether they find them or not at the clinic, let’s say they don’t find them and you may find that’s the only clinic in that area, but a mother in Serowe has many places to check from – Dietician*

*It all goes down to availability that is the biggest challenge … the demand is very high and there is no supply – Pharmacist*

*Currently the whole country is experiencing shortage of [some] medications. It has been awhile without medication including supplements. We are not the only ones who use these supplements, other people with other illnesses use supplements and this is a big problem – Reproductive health Officer*

Many participants noted a lack of communication from central medical stores on the reasons why the supplements were not available.

*There has not been any communication from the national level as to why they [iron and folic acid] are out of stock, not even an email to say we do not have these items. What we do is just order and the only time we realize they are out of stock is when we receive our packaging list, they will write not issued besides the tablets that are out of stock. – Pharmacist*

#### Competing priorities

The dieticians, pharmacists, and the reproductive health officers indicated that the government was not able to meet the demand for supplements as they gave priority to other orders over supplements. According to 1 pharmacist, supplements were competing with COVID-19 vaccinations and therapeutics. Over the past year, the focus has been on COVID-19 vaccination and less attention has been paid to medications like supplements, despite the high demand. A nurse/midwife also noted that priority is given to certain drugs like antiretroviral therapy for HIV and drugs to treat hypertension.

*Ever since Covid, priority has been Covid-related items… so they would have Covid drugs over the other medications, especially the supplements – Pharmacist*

#### Late antenatal care registration

However, healthcare providers indicated that some pregnant women registered late for antenatal care, limiting the window in which they could receive supplementation. The best time to register for antenatal care was said to be during the first trimester so that the woman could start supplements early in pregnancy.

*Pregnant women come [to the clinic] late, they maybe come after their first trimester and have missed the opportunity to take them immediately – Dietician*

#### Nonadherence and side effects

Nonadherence was considered another major challenge to daily supplementation during pregnancy. After collecting the supplements from clinics, healthcare providers indicated that some pregnant women put them aside and did not take them due to side effects or lack of knowledge about the benefits of supplementation. According to the dietitians in the study, some women were reluctant to take supplements due to side effects, including nausea, vomiting, and constipation, which were amplified by perceived bad taste or smell. Pharmacists and reproductive health officers also stated that some pregnant women were prescribed supplements but stopped taking them due to side effects.

*Sometimes the clinic has supplements, and we give them to the pregnant women, and they will be like “I’m sorry, but I cannot take these”…Some would say they smell bad – Reproductive health Officer*

Opinions about supplementation side effects were mixed. Although some providers were of the opinion that some supplements including iron caused nausea, vomiting, bad smell, and constipation, others stated that supplements did not have side effects and that nausea and vomiting were likely due to hormonal changes related to pregnancy.

Forgetting to take the supplements was also noted as another factor that impacted nonadherence.

*The most challenge we see is forgetfulness. You can give them tablets that would last for a month and when they come for their monthly check-up and you ask how many tablets are left-because that’s what we normally ask them, they will tell you that there is a lot of them left, and you ask why they are not taking them they would say it is because they forget or because of the side effects – Nurse/midwife*

#### Costs

Participants described that women are often encouraged to buy supplements at private pharmacies when supplements are unavailable at the clinics. However, the high cost of supplements at private pharmacies was noted as a challenge, and only some women can afford to purchase supplements at private pharmacies. Due to a struggling economy and high unemployment in the country, some pregnant women were said to not be able to buy nutritious foods and supplements. According to several participants, lack of money to buy supplements from the pharmacy was disadvantaging poor pregnant women.

*The only approach you can take is to advise those who can afford to buy to do so – Pharmacist*

#### Cultural and traditional beliefs

Participants indicated that cultural and traditional beliefs were sometimes drivers of nonadherence or not eating certain foods rich in iron and folate, such as eggs or liver. According to 1 nurse/midwife, some women did not eat foods rich in certain micronutrients like iron and folate as they had been advised not to eat them during pregnancy. To address this, 1 reproductive health officer indicated that it was important to address parents of the pregnant woman at the community level.

*Some do not take [supplements] due to their traditional beliefs, but we help them understand the benefits of supplements… Some would say my parents said I should not eat kidneys because my child would develop abnormalities, like big testicles. Some say when I eat liver, I would bleed a lot while giving birth and in cases like these we ask them to bring the parents to the next meeting, because the parent comes first so we can’t change what people have learned from their parents without involving them, so we sit them down and talk to them – Reproductive health officer*

### Domain 5: availability of foods rich in IFA and food fortification

All participants were asked about the availability of foods rich in IFA and feasibility and acceptability of food fortification. Generally, participants indicated that foods rich in iron and folate were available in markets and through backyard gardening, such as nuts, liver, beans, and leafy green vegetables. Nurse/midwives and reproductive health officers indicated that, in the absence of supplements, they encouraged women to eat foods rich in micronutrients. The dieticians echoed the need for women to eat a nutritious diet even if supplements were available because of the various nutrients in food as compared with supplements from tablets alone.

*From my side as a dietician I know that supplementation is good, but food is better. It’s the issue of availability, if I want to take a nutrient from food, the way it comes from food it’s not the only nutrient that I would gain but others too, but with supplementation, what I am supplementing is the only thing I will get, its specific, if I give a pregnant woman food rich in iron, I am able to achieve more concerning their health compared to the woman using supplements – Dietician*

In general, participants indicated that fortifying staple foods with micronutrients was acceptable and feasible, and some even indicated that fortified foods should be given to pregnant women due to frequent stockout issues.

*I believe that since there are foods already fortified like Tsabana and Malutu, because it has been done, there is nothing preventing us from doing it with other foods – Pharmacist*

*I believe it is highly possible looking at the needs, there are food programs that Botswana has… children collect Tsabana, so I think it can be extended to women of child-bearing age so they get the supplements – Pharmacist*

*Fortified food should be given to pregnant women but here it is not, where I used to work we used to give fortified food to all pregnant women, but here in clinic fortified food is not given to all pregnant women but only those with low weight – Nurse/midwife*

### Domain 6: supplementation use before pregnancy

All healthcare providers were asked about the feasibility and acceptability of supplementation before pregnancy. Several participants noted that this would be challenging given that many pregnancies are unplanned.

*This one is a bit of a challenge because according to my observation, most of the pregnancies are not planned so it is going to be difficult to say I’ll take supplements prior to pregnancy because most of the pregnancies are not planned, they only come forward when they are pregnant – Nurse/midwife*

Participants also noted that educating women on the benefits of supplementation before pregnancy would be essential to increasing uptake of supplementation before pregnancy. Currently, most education initiatives target pregnant women only, rather than all women of child-bearing age, and so many women only learn about the importance of supplements once they are pregnant.

*I think if women are educated and are aware of the benefits, I think they would accept it. It would be feasible. It’s a matter of them understanding that they need supplementation and why they need it. This can be done in a public health message so that when women plan to fall pregnant or when they fall pregnant, they are ready to supplement – Dietician*

The nurse/midwives also expressed that it was very rare for nonpregnant women to take supplements. One nurse/midwife noted there are no guidelines or policies on the provision of supplements before pregnancy, suggesting that the Botswana health protocol may not support supplementation for people who are not pregnant unless they have a medical condition. Nonpregnant women were said to be reluctant to take supplements when they were not suffering from any condition. One nurse/midwife indicated that young women associated taking supplements with pregnancy and so did not see the importance of taking the supplements before they became pregnant.

*I would say if we emphasized more on the benefits of taking the supplements, we would have more women taking them before they conceive, but the information is not reaching most people out there. That’s why it is not feasible. But if information [was provided] to the community, they will take them – Nurse/midwife*

## Discussion

We conducted 16 in-depth interviews of healthcare providers, including nurse/midwives, pharmacists, dietitians, and reproductive health officers, to assess perceptions and experiences of IFA supplementation before and during pregnancy in Botswana. These interviews captured experiences from a rural setting (Palapye) and an urban catchment area (Tlokweng). In our study, nurse/midwives demonstrated a high level of knowledge regarding the benefits of supplementation in pregnancy and prescribed supplementation to pregnant women when supplements were available. The major barrier to supplementation during pregnancy was availability, and many participants described frequent and long-lasting stockouts of supplements at antenatal clinics. Some individuals noted that stockouts may have been worse during the COVID-19 pandemic as more government funding went toward procuring COVID-19 therapeutics. Some of the other major challenges to adequate supplementation during pregnancy were late antenatal care registration, nonadherence and side effects, costs, and cultural and traditional beliefs regarding what foods should be eaten. Study participants generally agreed that foods rich in iron and folate were available, and that fortification of staple foods with IFA would be feasible and acceptable. However, providing supplementation before pregnancy was viewed to be challenging by many.

Our study team also conducted in-depth interviews with 20 pregnant women attending antenatal care at the same 2 clinics in Palapye and Gaborone, and results are reported elsewhere [30]. The healthcare provider interviews focused on clinical decision-making, prescribing practices, supply chain challenges, and systemic barriers to IFA supplementation, whereas the pregnant women interviews examined lived experiences, personal knowledge, adherence, and patient-level barriers, representing distinct analytical domains that warranted separate manuscripts. Similar themes were identified in interviews with pregnant women and interviews with healthcare providers. Similar to healthcare providers, pregnant women were knowledgeable about the benefits of receiving supplementation during pregnancy and were open to receiving fortified foods. Many of the same barriers to supplementation were identified in the interviews with pregnant women and with providers. Pregnant women also cited availability and stockouts as the biggest barrier to supplementation, and also described costs, side effects, and adherence as additional barriers. Although healthcare providers had mixed opinions about whether side effects should be attributed to supplementation or to early pregnancy, pregnant women were more likely to attribute side effects to iron supplementation. Both pregnant women and healthcare providers agreed that, to overcome these barriers, supplements should be made consistently available, health education could be improved, and nutritious and fortified foods could be supplied. Although healthcare providers questioned whether supplementation before pregnancy was feasible, most pregnant women were in favor of receiving supplementation with IFA before pregnancy.

Our findings are consistent with prior studies from sub-Saharan Africa and low- and middle-income countries demonstrating that inadequate micronutrient supplementation during pregnancy reflects a combination of supply constraints, insufficient counseling and maternal knowledge, concerns about side effects, and underutilization or late initiation of antenatal care services [[23], [24], [25], [26], [27], [28], [29],^34^]. Similar to these studies, we observed the importance of the role of health workers and antenatal counseling [25,27,35]. At the same time, our findings extend prior work by suggesting that supplement availability alone is insufficient, as counseling gaps and broader health system constraints can undermine uptake even when supplementation is available. For example, the COVID-19 pandemic also disrupted pharmaceutical medicines and micronutrient supply chains, further worsening access to maternal supplementation [[36], [37], [38]]. Furthermore, consistent with our findings, many studies have shown that nutrient fortified foods can be used as an alternative to specific micronutrient supplementation before and during pregnancy to prevent nutrient deficiency-associated complications. Mandatory folic acid fortification programs in the USA, Canada, Costa Rica, Chile, and South Africa are associated with significant increases in blood folate concentrations and declines of 25%–50% in the prevalence of neural tube defect-affected pregnancies [[39], [40], [41], [42], [43], [44]]. There are limited data on food fortification in Botswana; however, in 2021, the Food Fortification Initiative (FFI) partnered with the Botswana Government, Southern African Development Community (SADC), and the FAO of the United Nations to facilitate Botswana’s development of a national food fortification strategy. FFI began liaising with government stakeholders and SADC in 2019 to support the country’s micronutrient deficiency data needs to make a strong case for the implementation of mandatory fortification in the country [21].

Our results should be interpreted in the context of some limitations. Our study was conducted at 2 clinics in Botswana: Tlokweng and Palapye. Tlokweng was chosen to represent a more urban population, whereas Palapye serves more rural communities. The clinics are geographically distinct (Tlokweng is in the south whereas Palapye is in the north) and serve populations with diverse cultural practices and food preferences. As such, our findings may not be generalizable to other areas of Botswana, where perceptions and experiences with supplementation may differ. Additionally, carrying out this study during the COVID-19 pandemic may overemphasize realities on stockouts and financial strains, which may already be different as we emerge from this pandemic. Finally, there was a limited understanding of the specific supply chain processes for micronutrient supplementation in the country from providers, which may point toward engaging entities such as Central Medical Stores to better understand supply chain processes and challenges for guiding effective implementation strategies.

In conclusion, we found that healthcare providers in Botswana were knowledgeable and supportive of providing supplementation with IFA to pregnant women, but faced significant barriers to providing supplementation. There is an urgent need to improve micronutrient supplementation supply chain issues, especially during pandemics and other public health emergencies. Additional interventions to reduce barriers to micronutrient supplementation may include fortifying staple foods with folic acid, providing foods fortified with IFA to pregnant women, building strong primary care that could capture women capable of becoming pregnant to open up conversations around prepregnancy micronutrient supplementation, and educating providers on best strategies to address patient concerns around side effects of supplementation. Taken together with the findings from interviews with pregnant women, our study identified an urgent need to identify and evaluate interventions to increase the availability of micronutrient supplementation at antenatal clinics, with the ultimate goal of improving maternal and infant outcomes.

## Author contributions

The authors’ responsibilities were as follows – PK, RS, ECC: designed the study; PK: qualitative analyses were conducted; LM, PK, ECC: prepared the first draft of the manuscript; PK, RS, ECC, MD, RZ, GM, JM, MM, JM, SL, EL: participated in editing the manuscript and approved the final version for submission; and LM: had full access to all data and had the final responsibility for the decision to submit for publication.

## Data availability

The datasets used and analyzed during the current study may be available from the corresponding author on reasonable request.

## Funding

This work was supported by the National Institutes of Health NIH/NICHD K01 HD100222, NIH/NICHD R01 HD080471, NIH/NICHD K23 HD088230, NIH/NIAID K24AI131924.

## Conflict of interest

The authors declare that they have no competing interests.
