# Supplementary material for: Healthcare Providers’ Perceptions and Experiences of Prenatal Iron and Folic Acid Supplementation—A Qualitative Study in Botswana
Source: Curr Dev Nutr. 2026 Feb 25;10(3):107660. doi: 10.1016/j.cdnut.2026.107660 (PMC12991837; doi:10.1016/j.cdnut.2026.107660)
Supplement: Multimedia component 1 [file mmc1.docx]

**Supplementary Material for “Health care providers’ perceptions and experiences of prenatal iron and folic acid supplementation - A qualitative study in Botswana**

**Appendix 1. Semi-structured interview guides for interviews with healthcare providers.**

The following questions were used as a guide for interviews with nurses/midwives (N/M), pharmacists (P), dieticians (D), and sexual and reproductive health officers (SRHO). Questions asked only to nurses/midwives are indicated with (N/M only). All other questions were asked to all health care providers.

*Now we will start the interview. I will first ask you about indications and contraindications for prescribing iron and folic acid.*

**Section 1 (N/M only): Indications and contraindications for prescribing iron and folic acid**

*Question 1.1: Do you prescribe supplements with iron and folic acid to pregnant women? If yes, which ones? When in pregnancy do you prescribe them? If no, why not? If you only prescribe to some women, which women?*

*Question 1.2: Pregnant women are often prescribed iron alone, iron+folate, or a multiple micronutrient containing iron, folate and other micronutrients. What are the reasons why you would prescribe one versus another type of supplement?*

*Question 1.3: Do you prescribe supplements to women of child-bearing age who are not pregnant? Why or why not? If yes, which supplements?*

*We will now move on to talk about availability of supplements.*

**Section 2: Availability of supplements at clinic pharmacy and at national level**

*Question 2.1: In your experience, what supplements are available for pregnant women at antenatal clinic pharmacies?*

*Question 2.2: Which supplements are usually available and which are usually out of stock? If supplements are not available, do you know why?*

*Question 2.3: In your experience, what supplements are available at the national level?*

*Question 2.4: What other issues are there with supply and demand of supplements? Do you know when/where/how often supplements are available?*

*Question 2.5: If they are not available, do you know why?*

*We will now move on to talk about knowledge of supplementation.*

**Section 3 (N/M only): Knowledge of benefits of supplementation**

*Question 3.1: What are some of the benefits of supplementation with iron and folic acid during pregnancy?*

*Question 3.2: Do you discuss iron and folic acid supplementation with a pregnant woman at her first antenatal visit? If so, what information do you provide? Do you discuss this at the first visit only, or at each visit during pregnancy?*

*Question 3.3: Do you discuss foods that are rich in iron and folic acid with pregnant women at their antenatal clinic visits?* [Give examples].

*We will now move on to talk about side effects and adherence.*

**Section 4 (N/M only): Side effects of supplementation, delivery to reduce side effects, adherence**

*Question 4.1: Are there side effects from supplementation with iron and folic acid? If so, what are they? Do you discuss these with pregnant women?*

*Question 4.2: To your knowledge, how can side-effects be reduced? Do you discuss this with pregnant women?*

*We will now move on to talk about competing priorities to supplementation.*

**Section 5: Competing priorities to supplementation**

*Question 5.1: In your experience, what are the major challenges to daily supplementation during pregnancy in Botswana (probe for cost, other priorities, other reasons)?*

*Question 5.2: Are there any side effects of supplements? If so, which ones?*

*Question 5.3: In your opinion, are supplements accessible or not accessible to pregnant women in Botswana? Give reasons for your response.*

*Question 5.4: Are pregnant women knowledgeable or not knowledgeable about the benefits of supplementation? Give reasons for your response.*

*Question 5.5: What are some approaches to overcoming these challenges?*

*Question 5.6: What are the major challenges to daily supplementation prior to pregnancy?*

*We will now move on to talk about nutrition.*

**Section 6: Foods rich in iron and folic acid and food insecurity**

*Question 6.1: How available are foods rich in iron and folic acid in Botswana?* [Give examples]. *How does this vary by region or other factors?*

*Question 6.2: In your experience, how feasible/acceptable is fortification of staple foods with iron and folic acid in Botswana as a strategy for improving supplementation for women of child-bearing age?* [Give examples].

*Question 6.3: What are the key foods that could be fortified?*

*We will now move on to talk about supplementation before pregnancy.*

**Section 7: Vitamin supplementation prior to pregnancy**

*Question 7.1: In your experience, how feasible/acceptable/accessible is supplementation prior to pregnancy in Botswana? Does this vary by region or other factors?*

*Question 7.2 How common is it that a woman you see for the first time has been taking supplements at least somewhat regularly since she became pregnant? What about prior to pregnancy?*

*We will now conclude by talking about other barriers to supplementation.*

**Section 8: Other barriers and whether they vary by HIV status or other factors**

*Question 8.1: In your experience, what are other barriers to supplementation with iron and folic acid during pregnancy? Prior to pregnancy?*

*Question 8.2: Do these barriers vary by HIV status? By other factors? If so, how?*

*We have come to the end of the interview.*

*Thank you very much for your time.*
